# Densities and drivers of sea turtle populations across Pacific coral reef ecosystems

**DOI:** 10.1371/journal.pone.0214972

**Published:** 2019-04-24

**Authors:** Sarah L. Becker, Russell E. Brainard, Kyle S. Van Houtan

**Affiliations:** 1 Monterey Bay Aquarium, Monterey, California, United States of America; 2 NOAA Pacific Islands Fisheries Science Center, Ecosystem Sciences Division, Honolulu, Hawaii United States of America; 3 Nicholas School of the Environment and Earth Sciences, Duke University, Durham, North Carolina United States of America; University of Plymouth, UNITED KINGDOM

## Abstract

Sea turtle populations are often assessed at the regional to sub-basin scale from discrete indices of nesting abundance. While this may be practical and sometimes effective, widespread in-water surveys may enhance assessments by including additional demographics, locations, and revealing emerging population trends. Here, we describe sea turtle observations from 13 years of towed-diver surveys across 53 coral islands, atolls, and reefs in the Central, West, and South Pacific. These surveys covered more than 7,300 linear km, and observed more than 3,400 green (*Chelonia mydas)* and hawksbill *(Eretmochelys imbricata*) sea turtles. From these data, we estimated sea turtle densities, described trends across space and time, and modelled the influence of environmental and anthropogenic drivers. Both species were patchily distributed across spatial scales, and green turtles were 11 times more abundant than hawksbills. The Pacific Remote Island Areas had the highest densities of greens (3.62 turtles km^-1^, Jarvis Island), while American Samoa had the most hawksbills (0.12 turtles km^-1^, Ta’u Island). The Hawaiian Islands had the lowest turtle densities (island ave = 0.07 turtles km^-1^) yet the highest annual population growth (*μ* = 0.08, *σ* = 0.22), suggesting extensive management protections can yield positive conservation results. Densities peaked at 27.5°C SST, in areas of high productivity and low human impact, and were consistent with patterns of historic overexploitation. Though such intensive surveys have great value, they are logistically demanding and therefore have an uncertain budget and programmatic future. We hope the methods we described here may be applied to future comparatively low-cost surveys either with autonomous vehicles or with environmental DNA.

## Introduction

Sea turtles are highly migratory species whose complex spatial population structures challenge management and conservation efforts [1, 2]. Although life history patterns are broadly defined, much about the demographics, abundance, and distribution of Pacific populations remains unresolved. Clarifying these unknowns has been limited by obstacles inherent to sea turtle ecology. Breeding, nursery, and foraging habitats are often widely-dispersed, geographically-discrete sites [3, 4]. Another complication is that sea turtles may take several decades to mature, then only return to breeding grounds every several years [1, 5–7]. Their vast ranges and lengthy life histories require unusually large-scale monitoring efforts. In the Pacific, many of the numerous and isolated archipelagoes are regularly visited by researchers for coral reef surveys, presenting an opportunity to observe local turtle populations.

Green and hawksbill sea turtles are commonly associated with coral reef habitat. Both are historically exploited species whose populations still remain endangered today [8, 9]. Despite decreases in harvest with protective measures initiated in the 1970s, current populations are still at a fraction of historic levels [10]. Listed as critically endangered by the IUCN and as endangered throughout its range by the U.S. Endangered Species Act, hawksbill turtles are the most at risk of the globally-distributed sea turtle species [8]. Green turtles are also listed as endangered by IUCN [9], though more recent assessments documented significant population growth trends and improved conservation status [7].

The legacy of exploitation is directly associated with human population density [11]. Hawksbills were largely exploited for tortoiseshell, while greens were predominantly killed for consumption [10]. Both species are still at risk from continued harvest, fisheries bycatch, ocean plastics and pollution, and habitat loss and degradation [1, 5]. In addition to direct mortality from human impacts, the survival and recovery of sea turtles is limited by the sensitivity of coastal habitats to environmental and anthropogenic stressors. Coral reefs, an important feeding ground for green and hawksbill sea turtles, are highly sensitive to and threatened by overfishing, terrestrial runoff, and climate change [12, 13].

Population monitoring is vital to determine population threats and evaluate conservation management strategies. Nearly all basin-level assessments of sea turtle distributions have focused on nesting locations [6, 7], biasing ecological understanding in favor of sites and habitats used for breeding. Though more logistically challenging and expensive, visual in-water surveys provide a more complete representation of sea turtle population distribution. By broadening the monitoring efforts to include foraging sites, such surveys could improve quantification of adult recruitment, fitness, and survival [5, 14]. Incorporating multi-taxa surveys into preexisting monitoring programs could provide vital in-water data while distributing the logistical burden of large-scale observational studies across diverse programs and funding streams.

Density, which reflects the interplay between reproduction, resource availability, behavior, and top-down forces, is widely used to compare populations across time and space. Relative density allows for comparisons regardless of sample area, making it a useful and commonly applied metric. This study utilized 13 years of in-water survey data of green and hawksbill sea turtles from NOAA’s Pacific Islands Fisheries Science Center for 53 islands, atolls, and reefs throughout the U.S. Pacific. These data are the first comprehensive in-water surveys for the region, and for many sites the first in-water sea turtle surveys ever conducted. To summarize population trends across the U.S. Pacific and assess the distributions of each species, we illustrated demographic patterns among species and islands and quantified density at region, island and yearly scales. Using the density values, we compared between species, locations, and across time, identified regions of high and low density, and quantified yearly population growth rates. We also examined potential environmental and anthropogenic drivers of these population trends by modelling the influence of sea surface temperature (SST), productivity, habitat area, and human impacts on observed variation in turtle density.

## Materials and methods

### Towed diver surveys

The Coral Reef Ecosystem Division (CRED) at NOAA’s Pacific Islands Fisheries Science Center conducted biennial or triennial nearshore surveys of coral habitat from 2002 to 2015 during the month of April. CRED surveyed 53 sites consisting of islands, atolls, and reefs in four regions of the U.S. Pacific Islands: American Samoa (AMSM), the Hawaiian Archipelago (HIIS), the Mariana Archipelago (MARI), and the Pacific Remote Island Area complex (PRIA). During surveys, a boat towed two SCUBA divers at slow speed (~1.5 kts or 2.6 km/hr^-1^) and consistent depth (~15 m). Divers operated specialized dive planes (often termed “towboards”), recording benthic habitat and population observations of predominantly corals, fish, and other marine life along a 10-meter wide path throughout forereef habitat [15]. Additionally, divers recorded all observations of turtles and other megafauna within visual range of the transect. Surveys consisted of a series of 50-minute tows covering ~2.2 km, where each tow was divided into ten 5-minute segments covering ~220 m. In each tow segment, divers recorded the date, location, number of turtles seen, species (green, hawksbill), and estimated average total turtle length. Turtle sex was not identified. Further details describing these methods are provided elsewhere [16].

### Turtle demographics

We calculated the relative population proportion of life history stages (new recruit, juvenile, subadult, and adult) for both species at each site. Life stages were determined by observer turtle-length estimates and previous empirical studies that determined length-to-age groupings. The straight carapace length (“SCL”) for each turtle was L*0.8, where *L* is the observed length. For green turtles, new recruits were < 35 cm, 35 ≥ juveniles < 65 cm, 65 ≥ subadults < 80 cm, and adults were ≥ 80 cm [17]. Hawksbill classes are slightly smaller with new recruits < 35 cm, 35 ≥ juveniles < 58 cm, 58 ≥ subadults < 72 cm, and adults ≥ 72 cm [18, 19]. As early observations had unassigned species identifications (~1% of raw data), we assigned identifications to these observations based on the later multi-year species proportions for each location. We limited demographic characterization to locations with ≥ 20 observations per species.

### Estimating population density and growth

To describe population density, we fit discrete, non-negative distributions to the raw turtle count data. For each site surveyed on > 2 occasions, we fit Poisson, negative binomial, zero-inflated Poisson (ZIP), and zero-inflated negative binomial (ZINB) models to the data. The R package ‘fitdistrplus’ [20] fit the Poisson, negative binomial, and ZIP models, and the ‘MASS’ package [21] fit the ZINB. The ‘gamless’ package [22] defined density functions for ZIP and the ‘emdbook’ package [23] for ZINB. Fitted models estimated the probabilities of observing 0–22 turtles, the range of turtles observed per segment. AIC ranked the negative binomial highest for the majority of sites or was within 3 AIC units (see Results) therefore we used this model to examine all subsequent turtle densities in space and time.

Fitted models calculated relative turtle densities. We used the fitted parameters for each site to calculate the turtles observed given standard effort (1000 tow segments). We performed this routine for all species combined, by species, by site, and then by survey year. To visualize basin-level patterns, we mapped mean turtle densities at each site over all study years. We assessed temporal trends at the regional scale, comparing the density at each site and year. Boxplots summarize annual density at the regional level. Over each regional series, we plotted a locally-weighted regression (“LOESS” [24]). Predicted values from the LOESS determined the annual rate of change. Assuming stochastic exponential growth [6, 7], we natural log-transformed predicted LOESS values for surveyed years and calculated the rate of change (*r*_x_) between each consecutive year of predicted LOESS values. The mean (*μ*) and standard deviation (“sd”, *σ*) of *r*_x_ summarized population growth.

In addition to comparing density, we estimated the total sea turtle population abundance at each site and region. We expanded the fitted densities given the survey effort at each site (determined as the mean number of tow segments across all survey years). As tow segment lengths are regulated by time, we used the average segment length at each site to convert catch per unit effort density into spatial density (turtles km^-1^, see Table A in S1 Appendix). We then predicted the cumulative turtles per survey year to estimate regional abundance.

### Explaining turtle density

**Fig 1:**
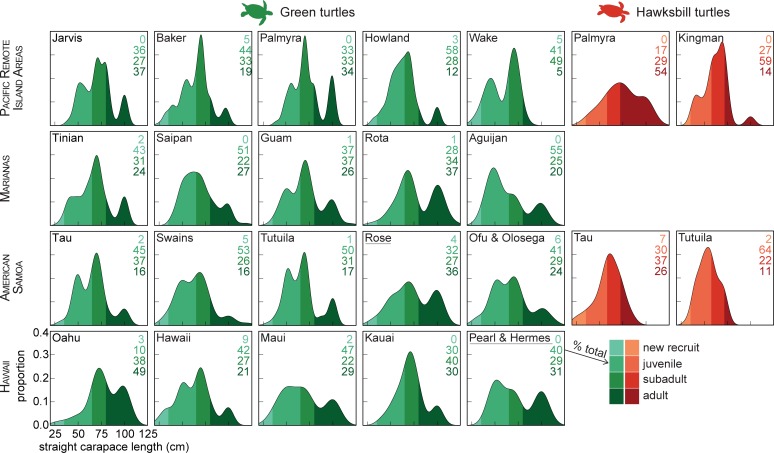
For most sites, observed abundance decreases with size class. We observe that juveniles are most common, followed by subadults, and adults. New recruits are rare. Notable exceptions, are the dominance of green turtle adults on Rota (37% of surveyed turtles), Rose Atoll (36%), and Oahu (49%), and adult hawksbills at Palmyra Atoll (54%). Though low-level nesting can be widespread, for both species, only Rose Atoll and Pearl & Hermes (underlined labels) are documented nesting sites (> 30 nesters per season) in these regions. For green turtles, remote Wake Atoll had the lowest proportion of adults (5%), Hawaii Island had the largest proportion of new recruits (9%). Ta’u had the most hawksbill new recruits (7%). Smoothed histograms show the proportion of turtles in each size class, calculated from diver estimates of straight carapace lengths. Histogram and label colors saturate as size class increases. Labels give the percentage of each class, with the most abundant size classes in bold. Only sites with > 20 turtles of a given species observed over all survey years were analyzed.

We modelled variation in sea turtle density across sites using random forest (“RF”) models and assessed model sensitivity with a leave-one-out cross validation (“LOOCV”) using the ‘randomForest’ [25] package in R. We constrained this analysis to green turtles, due to the low abundance and restricted distributions of hawksbills (see Fig 1). The RF model included available neritic habitat area (km^2^), sea surface temperature (“SST”,°C), productivity (mg chlorophyll-a m^-3^), and cumulative human impact score [26]. Each model run built 500 trees and tested 2 predictors at each node (mtry = 2). To calculate the available neritic habitat metric we used fine-scale bathymetry maps generated by the Pacific Islands Benthic Habitat Mapping Center (PIBHMC) and the Hawaii Mapping Research Group (University of Hawaii, SOEST) to quantify area of < 30 m depth surrounding each island. Fine scale bathymetry data were available for 35 of the 53 surveyed sites, and only these locations were included in the analysis. We derived SST from monthly Aqua/MODIS records (Sea Surface Temperature 1 month—Aqua/MODUS) for the month of April from 2003–2015: the same timing of the surveys. Monthly Aqua/MODIS data also provided chlorophyll-a (“chl-a”) concentrations (Chlorophyll Concentration 1 month—Aqua/MODIS) for all months from 2003–2015. Cumulative human impact was defined as the average of the Halpern and Fujita [27] index for a 100-km radius surrounding the centroid of each island.

We generated variable importance (mean decrease in MSE under permutation), training and test RMSE, and adjusted *r*^2^ for each LOOCV iteration. To assess variable influence, model performance, and model sensitivity, we calculated the mean and sd of each metric. After assessing model sensitivity and fit with LOOCV, we trained a global model on the full dataset to describe trends for each variable. We visualized the raw relationships between drivers and densities, and using the ‘pdp’ R package [28] we plotted individual conditional expectations (“ICE”) and partial dependent plots (“PDP”) for paired relationships from the RF model. All analyses were conducted within the R environment (R Core Team 2018) and all visualizations were generated in the R package ‘ggplot2’ [29].

## Results

### Relative species abundance

Across all the surveyed sites, green turtles represented 90.1% and hawksbills 8.3% of observations, with the remaining 1.6% unidentified. This suggests that green turtles are nearly 11 times more abundant than hawksbills across the entire survey area, providing further empirical evidence of the rarity and conservation plight of hawksbills [8, 18, 30].

### Demographics and model fitting

Observed age structure followed the commonly described pattern [5] of mostly juveniles, fewer larger age classes, with new recruits being fewest (Fig 1). For both species, adults were the largest age class at 25% of islands. Juveniles were clearly dominant for green turtles at 60% of locations, subadults were most common at 50% of the hawksbill locations (Fig 1).

**Fig 2:**
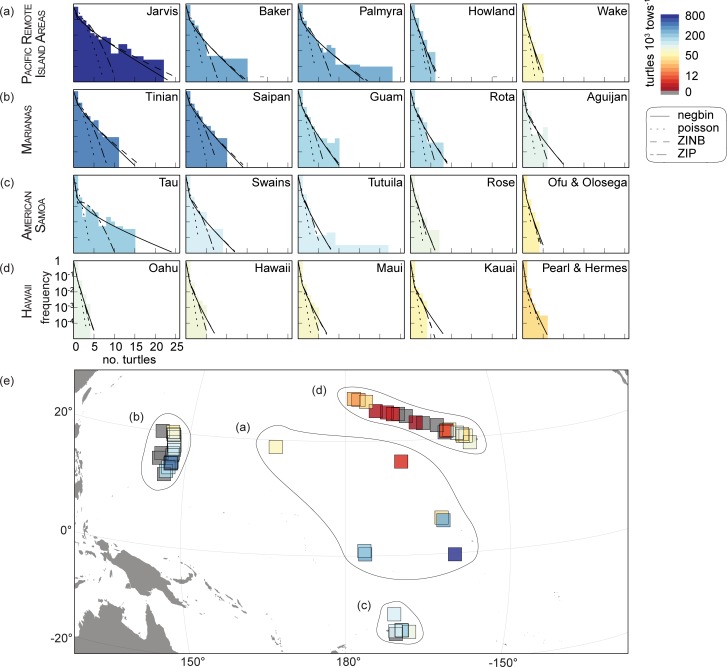
Densities and distributions of sea turtles in the U.S. Pacific Islands. The greatest sea turtle density is in the (A) Pacific Remote Island Areas (268 turtles per 1000 tow segments), followed by (B) the Mariana Archipelago (127), (C) American Samoa (93), and (D) the Hawaiian Archipelago (27). Jarvis Island had an astonishing density of 844 turtles; more than twice the density of any other site. Negative binomial models were consistently the highest-ranked across all sites, best describing the zero-inflated, heavy-tailed data. Histograms are shaded according to the modeled site-level density. Where the above panels model the densest turtle populations in each region, (E) maps the densities for all sites. The protected and remote Northwestern Hawaiian Islands have a surprisingly low sea turtle population densities. Squares show sites shaded by modeled density per 1000 tow segments (10 m x ~220 m).

Turtle observations were zero-inflated for all sites, with certain sites having localized clusters of high abundance (Fig 2A–2D). The fitted models indicated the probability of observing turtles steeply declined with increasing distance from the origin (i.e. 0 turtles). For many sites, ≥ 5 turtles were never observed in a single segment. Other sites had heavy-tailed distributions with as many as 22 turtles in a single segment (Fig 2A–2C), albeit at low probabilities. AIC rankings showed that the negative binomial was the highest-performing model (ΔAIC always ≤ 2.4, see Table B in S1 Appendix) simultaneously capturing the predominantly low-density and occasional high-density segments across sites. The ZINB was the next highest-ranked model, displaying a similarly robust fit. However, due to the zero-inflation parameter the ZINB rank suffered an additional penalty in AIC estimates.

### Turtle densities

The remote PRIA had the greatest turtle densities across all regions with 268 (± 290) turtles per 1000 tow segments. This was followed by MARI with 127 (± 109) and AMSM with 93.1 (± 58.4) turtles per 1000 tow segments. HIIS had the lowest observed densities, averaging just 26.9 (± 23.1) turtles per 1000 tow segments. Density mean and density SD at the regional scale were positively correlated, indicating patchiness and heterogeneity. Densities in PRIA, for example, ranged from 5–843 turtles per 1000 tow segments, while the low numbers across HIIS were more consistent, ranging from 0–74 turtles per 1000 tow segments (Fig 2).

Individual high-density sites strongly influenced regional summaries. Jarvis Island had the single highest site densities: 844 turtles per 1000 tow segments for combined species and 822 green turtles per 1000 tow segments. This number was > 2 times the next densest site. Tinian (394 turtles, 392 green turtles per 1000 tow segments) and Saipan (344 turtles, 344 green turtles per 1000 tow segments)–both < 50% the density of Jarvis–were themselves more than twice as dense as all other sites within MARI. Johnston Atoll (5 turtles, 5 green turtles per 1000 tow segments) had extremely low densities by comparison to other PRIA sites, but similar values to those in HIIS, to which it is in much closer proximity. For 13 sites in HIIS, AMSM, and MARI, 0 turtles were detected over all surveys (see Table A in S1 Appendix).

**Fig 3:**
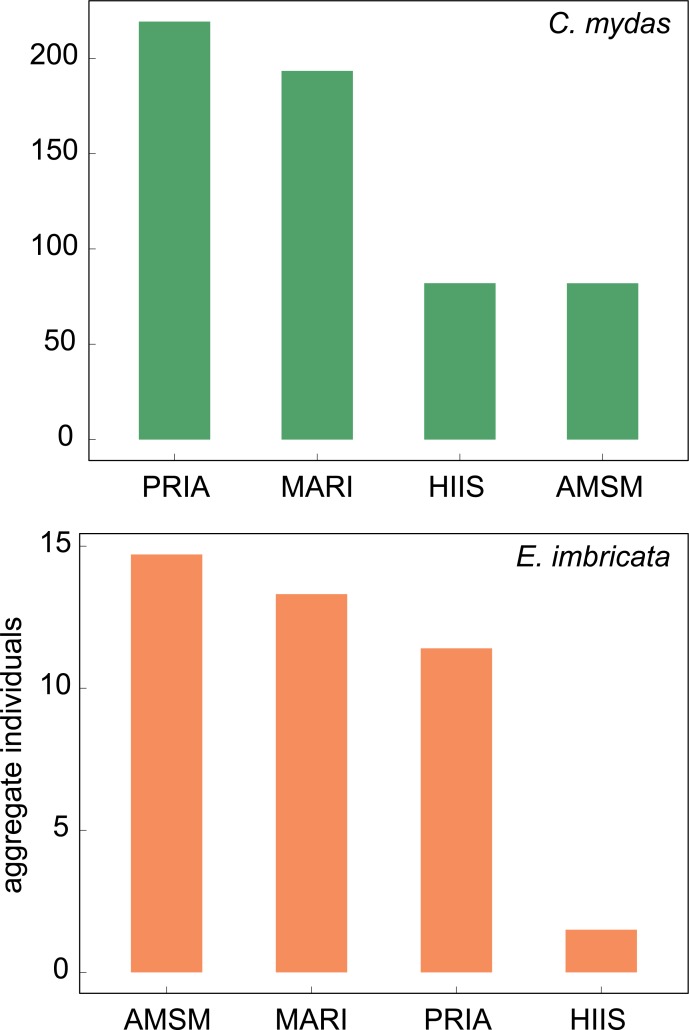
Predicted number of individual turtles detected in surveys varied by species and region. Density models predicted > 14 times as many green turtles (576) as hawksbills (41) predicted across all regions: the Pacific Remote Island Areas (PRIA), American Samoa (AMSM), the Mariana Archipelago (MARI), and the Hawaiian Archipelago (HIIS). PRIA had the greatest number of green turtles (219), followed by MARI (193), HIIS (82), and AMSM (82). While densities were far greater for PRIA than MARI, number of green turtles were similar. For hawksbills, AMSM had the greatest number (15), followed by MARI (13), PRIA (11) and HIIS (2). Higher abundance of detected green turtles in the PRIA and hawksbills in AMSM occurred despite the relatively few number of sites surveyed for each of these regions. Correspondingly, HIIS showed low numbers despite more sites, substantially larger available habitat area, and a greater total survey distance than all other regions (HIIS: 772, MARI: 364, PRIA: 286, AMSM: 257 km). We multiplied densities per site (Fig 2) by average number of tow segments per annual survey to calculate predicted individuals per site, then summed all site values for each region for aggregate predicted individuals. Green turtles are shown in green, and hawksbills in orange.

Consistent with density results, PRIA had the greatest aggregate predicted abundance of green turtles (219), followed by MARI (193), AMSM (82), and HIIS (82, see Fig 3). AMSM had the greatest hawksbill abundance (15), then MARI (13), PRIA (11), and HIIS (2). Importantly, aggregate sea turtle abundance did not stem from greater survey lengths. PRIA and AMSM showed high abundance for greens and hawksbills respectively despite small surveyed areas (PRIA: 286 km, AMSM: 257 km). MARI had the second highest abundance of both species over a survey distance of 364 km. HIIS, despite having double the surveyed distance (772 km), demonstrated low predicted individuals for both species. Regionally, PRIA was the only region with sea turtles observed at all sites.

### Trends through time

**Fig 4:**
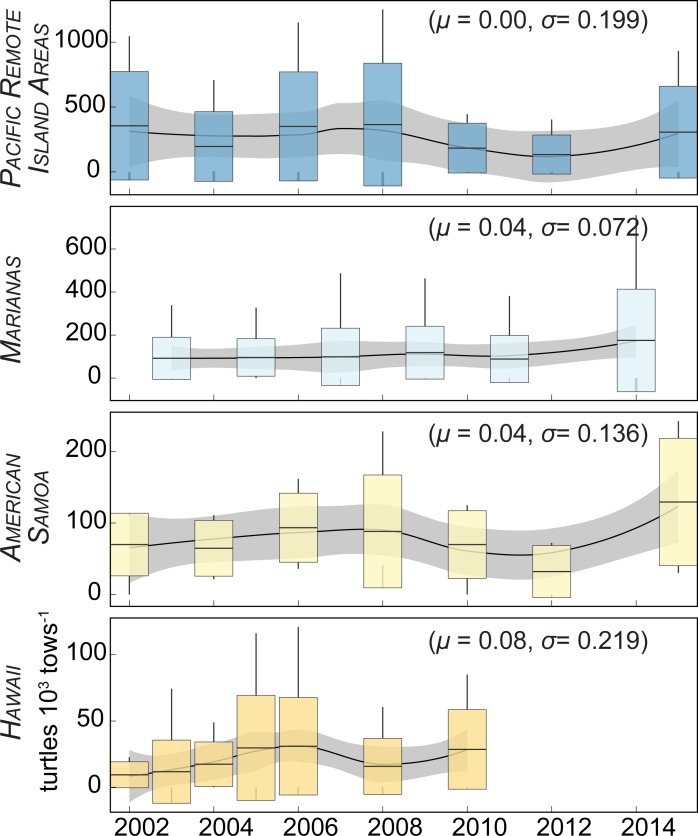
From 2002–2015, regional green turtle densities were stable or increased. Box plots (mean, 1 sd, min-max) show fitted density estimates for sites in each region, by year. All regions show growth from 2002–2008, declines from 2008–2012, and growth thereafter. From these times series, stochastic exponential growth models calculate rates of change. We list the fitted parameters. Population growth rate was inversely correlated with population size, which though not unexpected, may indicate density dependence. Black line is a LOESS model with standard error (gray band).

Regional trends through time indicated turtle densities were stable to increasing (Fig 4). Annual growth from 2002–2015 had an inverse relationship with density. HIIS had the highest mean annual growth rate (0.08 ± 0.219), while PRIA had the lowest growth (0.00 ± 0.199). All regional mean densities fluctuated over time—displaying a general pattern of growth from 2002–2008, followed by a slight decrease over 2008–2011, and subsequent increase. MARI and AMSM, in particular, had similar mean annual growth rates. For all regions except PRIA, the most recent surveys indicated the highest densities observed. Constraining these comparisons is that the HIIS surveys, unlike other regions, ended in 2010.

### Environmental drivers

**Fig 5:**
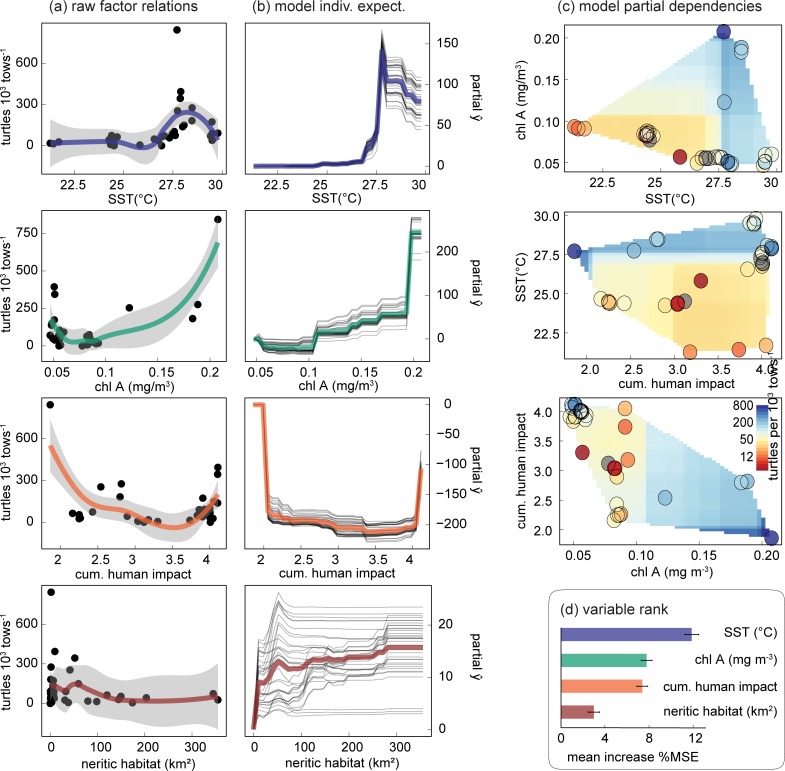
SST and productivity are the highest-ranked drivers of sea turtle densities. We used RF models to assess how SST, ecosystem productivity, available habitat, and human impacts affected sea turtle densities. (A) Scatter plots of single factors reflect expected relationships. Densities show a thermal envelope peaking at 27.5°C, increase with ecosystem productivity, decrease with an index of human impact, and surprisingly show little effect from habitat area. Filled circles are site densities, colored lines are LOESS fits (gray bands are standard error). Results from the RF model formalize these relationships in (B) ICE plots and (C) two variable PDPs. Shaded polygons represent the predictor space, while filled circles show the observed density values. The modeled habitat-density effect now appears positive, but noisy. Partial ŷ values assess the average change in the predicted value from each model factor, thin gray lines represent each site, and colored lines are the mean relationship. For some sites, (C) the negative influence of human impacts to turtle density appears mitigated by optimal SST. (D) Model variables are ranked according to the percent increase in MSE of removing each variable. The average was taken across each iteration of a LOOCV. Historical overexploitation, conservation efforts, and spatial population structure likely contribute to the unexplained error, and quantifying these factors may improve model performance.

The RF model detected expected relationships in the modelled drivers, but overall model performance was low (Fig 5D). The mean validation adjusted *r*^2^ across all runs of the LOOCV was 35.6% ± 0.06. LOOCV training RMSE was 52.2 ± 10.5, while validation RMSE was 123.79 ± 11.09. Mean variable importance across the LOOCV (Fig 5D) listed SST as the most influential driver (11.87 ± 0.63% increase in MSE), followed by chl-a (7.78 ± 0.49%), human impacts (7.4 ± 0.49%), and lastly neritic habitat area (3 ± 0.52%).

SST was the most influential driver of turtle density. Turtle density peaked at 27.5°C in both the exploratory pairwise comparisons and modelled partial dependency plots (Fig 5A–5B). The lowest observed density occurred mostly in the low SST range (in the Hawaiian Islands). Chl-a showed a positive relationship, and human impacts showed a predominantly negative relationship with density. However, densities increased at the highest levels of human impact, possibly indicating an interaction or unmeasured driver. Partial dependency plots showed a comparatively weak mean positive trend between neritic habitat area and density. Two-way PDPs illustrated possible interactions between the three most important variables (SST, chl-a, and human impacts) and showed that some islands with high densities had high human impact and low chl-a but ideal SST, reinforcing SST as the primary driver (Fig 5C).

## Discussion

Broad differences in densities across sites and regions provided a valuable framework to understand drivers of sea turtle abundance. Our modeling of these consistent and widely-distributed observations confirmed the well-known scarcity of the hawksbill sea turtles across a broad portion of the Pacific [8, 19, 30] and identified AMSM as a population of significance for hawksbills. Hawksbills were heavily exploited historically for tortoiseshell [8, 30] and of all the turtle species are most closely tied with highly-threatened coral reef habitat on which they depend for sponges and invertebrate prey [31]. Though widely distributed amongst the sites surveyed, green turtle abundance varied between sparse populations at some sites and other sites where they were observed in relatively high densities.

The zero-inflated and heavy-tailed nature of observations we documented here has been demonstrated across a variety of ecological settings [32, 33]. This patchy distribution of turtles occurred within as well as between islands, suggesting that there were both local and regional influences on turtle density. Low densities across HIIS are perhaps surprising given the distance from major human populations, extensive protected area in the Northwestern HIIS, and significant conservation investments over the past several decades. Densities in MARI, on the other hand, were greater than expected given the high degree of human impacts [27, 34]. Population growth trends were inversely correlated with regional density, though site variability within each region was high. This inverse relationship suggests that populations at lower impacted areas, such as PRIA, may be at equilibrium [35]. These higher density regions have historically had low human densities and likely low rates of historical exploitation. Their turtle populations may therefore be closer to pre-exploitation levels than regions with a stronger legacy of harvest [30, 36]. Correspondingly, low density regions with higher growth rates such as HIIS may indicate population recovery from historic exploitation.

RF models identified SST as the most influential factor in our model. This bottom-up driver describes peak green turtle densities occurring at 27.5°C. The importance of SST is consistent with the documented influence of SST on sea turtle population dynamics [37–40]. This could represent an ideal window of metabolic efficiency [41] or thermoregulation [42] for green turtles, or could indirectly drive significant ecosystem features such as foraging habitat. Interestingly, a similar 27–28°C occurrence peak exists globally for commercial fish species([43] Figure E in S1 Appendix). As our surveys and SST values only reflect April populations and temperatures, it would be valuable to track seasonal temperature changes and document any corresponding shifts in sea turtle densities. Given the importance of SST in our model, it is worth considering how warming might shift turtle densities. If documented rates of warming continue [42], HIIS, with the coldest SST and densities observed here might see greater turtle population growth, while some other areas might surpass the optimal thermal window of 27.5°C. Intense equatorial and topographic upwelling at the equatorial PRIA (Jarvis, Howland, and Baker) may keep these highly productive locations relatively cooler even under warming ocean conditions. Other environmental and anthropogenic drivers showed expected (but low-influence trends), with high densities positively correlated with high chl-a, low human impact, and very weakly with available habitat area.

The model error may be explained by several variables, some of which are inherently difficult to quantify. Ocean currents are influential in facilitating natal and breeding migrations and driving the locations of breeding sites [44–46]. We initially incorporated satellite-derived current amplitude (OSCAR third degree resolution ocean surface current currents. Ver. 1) in the model, but the model performed the same without it. Historical overexploitation is known in areas we found to have low density [36, 47], but a wide assessment of historical harvests is not available. Marine protected areas are not evenly distributed across the region and though they may be locally significant [48] their enforcement is not consistent [1]. The spatial population structure for many sea turtles, even green and hawksbills, is not well resolved [49]. While our model showed that neritic habitat area alone did not impact densities to a high degree, site-scale benthic habitat data were not available for many sites within this study. Though outside the scope of this study, fine-scale variations in habitat and community such as depth, reef complexity, food availability, habitat function, and predation risk also influence localized abundance. Future studies may improve upon our analyses by filling the gaps in several of these influential data streams.

Jarvis Island, a remote oceanic pinnacle with strong equatorial and topographic upwelling, drove many of the observed model relationships. An influential outlier (Fig 5A–5B) with densities > 4 times any other site (Fig 2A), Jarvis is noted for its placement in the equatorial undercurrent (EUC). This results in strong, but temporally variable, localized topographic upwelling on the west side of the island as the subsurface EUC encounters the submarine flanks of the island [50], causing Jarvis to have the highest chl-a concentrations among our study locations. However, the true scale of this productivity hotspot may be muted within our island-scale analysis. Beyond productivity, the SST of Jarvis Island was optimal and Jarvis had the lowest human impacts. Howland and Baker Islands, located 1000 km west of Jarvis, also experience both topographic and equatorial upwelling, but the intensities of both are reduced. Corresponding densities at these two sites are higher than most other islands, but still < 50% of that seen at Jarvis.

Though filling vital data gaps in comparison to nesting surveys, towed diver surveys have some inherent caveats. Detectability is influenced by sampling structure and timing, and while multi-taxa surveys are efficient, they could be suboptimal for any one taxa. In addition to identifying avenues for improving model performance, the methodologies that were originally developed for monitoring fish could be tailored more specifically to turtle sightings. The study presented here covers two species and multiple life stages. While there are not large differences in detectability between species or age class, some bias does exist within these data. Backreef lagoons are often important foraging habitats for green turtles [51], but the NOAA coral reef surveys focused almost exclusively on forereef habitat due to logistic constraints [16, 52]. Incorporating fine-scale benthic habitat data would also inform the ecological function of high-density regions. Structuring sampling in proportion to available turtle habitat would allow for transects to be used to estimate abundance rather than the relative density and abundance per survey area metrics utilized in this study.

Sampling across seasons could limit bias caused by breeding migrations. April may overlap with some breeding in the northern hemisphere, and demographics may therefore be underestimating adults in foraging areas. However, as most analysis in this study were not demographic specific and juveniles and subadults more common, comparative densities and abundance values presented here are viable. As such logistically-intensive operations are increasingly replaced by autonomous underwater vehicle and environmental DNA surveys [53, 54], both efficiency and utility may be improved. Reducing the logistical burden of monitoring can create new opportunities to survey larger areas and remote locations in a more flexible framework. This flexibility is needed in order to address large scale issues of monitoring and global change, as well as, fine-tune sampling to answer more nuanced questions. Though the existing data streams we explore here have great value for monitoring sea turtle population (Figs 1–5), they are ship-based and therefore require a significant capital support [55]. As such budgetary and programmatic support is not guaranteed, and in fact has declined, exploring whether autonomous survey platforms or environmental DNA surveys [56, 57] could be of similar value is worth exploring.

Understanding the relationships between turtle populations and environmental drivers will help managers predict and protect turtle populations given rapid global change. The sites covered in this study display varying amounts of isolation and differing degrees of cross-jurisdictional management, which introduces additional complexity when developing strategies to enhance population recovery. HIIS is the only region in this study where there is full overlap in population structure and political boundaries. [49]. Hawaiian green turtles are considered a genetically distinct population whose full life history occurs within HIIS, creating the potential for cohesive management [9, 49]. However, many conservation and management challenges still exist. HIIS is highly isolated, has high rates of historic exploitation, and has the coldest mean SST across this study. AMSM and MARI also have high rates of historic take and current human impact in the populated islands, but may currently have more favorable environmental conditions. PRIA, with the highest densities, are currently and formerly the least impacted by human exploitation, and certain areas exhibit high upwelling-driven productivity. All regions besides HIIS, however, may be linked via breeding migrations to highly threatened areas [9]. Temporal patterns show the potential of formerly high-exploitation sites with low sea turtle densities to rebound under strict enforcement, but bottom-up environmental forces may limit this recovery.

## Supporting information

S1 Appendix Supporting information for Densities and drivers of sea turtle populations across Pacific coral reef ecosystems. Fig A. Species proportions for each survey year for each island demonstrate greater abundance of green sea turtles. Fig B. Count frequency histograms and model fits for all sites not included in the main manuscript. Fig C. Island scale annual turtle densities show variable trends between survey locations and species. Fig D. Random forest model contains a great deal of unexplained variation, particularly for islands with high turtle densities. Figure E. The climate envelope of 993 exploited marine fishes peaks at 27–28°C, similar to our observation for sea turtle density (Fig 5A–5B). Table A. Density and predicted number of individuals per survey area. Table B. ΔAIC values for towed-diver observations for each study location fit with four candidate models. Table C. Survey effort by location.
(DOCX)
