# Supplementary material for: Densities and drivers of sea turtle populations across Pacific coral reef ecosystems
Source: PLoS One. 2019 Apr 24;14(4):e0214972. doi: 10.1371/journal.pone.0214972 (PMC6481790; doi:10.1371/journal.pone.0214972)
Supplement: S1 Appendix — Fig A. Species proportions for each survey year for each island demonstrate greater abundance of green sea turtles. Fig B. Count frequency histograms and model fits for all sites not included in the main manuscript. Fig C. Island scale annual turtle densities show variable trends between survey locations and species. Fig D. Random forest model contains a great deal of unexplained variation, particularly for islands with high turtle densities. Figure E. The climate envelope of 993 exploited marine fishes peaks at 27–28°C, similar to our observation for sea turtle density (Fig 5A–5B). Table A. Density and predicted number of individuals per survey area. Table B. ΔAIC values for towed-diver observations for each study location fit with four candidate models. Table C. Survey effort by location. (DOCX) [file pone.0214972.s001.docx]

Supplementary Materials for

**Densities and drivers of sea turtle populations across Pacific coral reef ecosystems**

Sarah L. Becker,^1^* Russell E. Brainard,^2^ and Kyle S. Van Houtan ^1,3^

* Corresponding author

Email: sarah.becker3@gmail.com

**
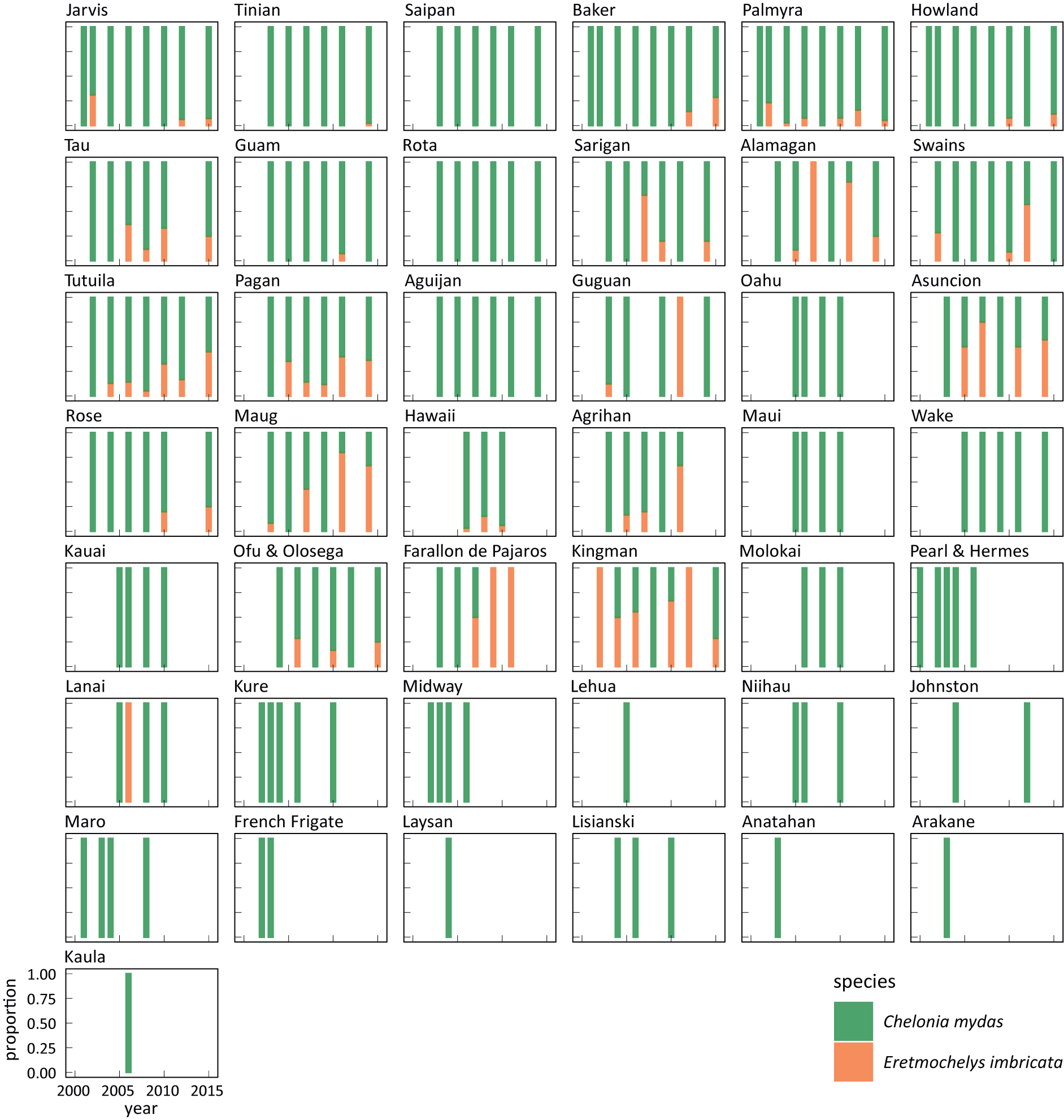
**

**Figure A.** **Species proportions for each survey year for each island demonstrate greater abundance of green sea turtles (*Chelonia mydas*).** Green proportions are higher in most locations, though several islands show approximately equal or greater proportions of hawksbills (*Eretmochelys imbricata*). Panels are arranged in order of greatest to least turtle density. 1.6% of raw data did not include species ID. In these cases, species ID was assigned based on species ratios for each island.


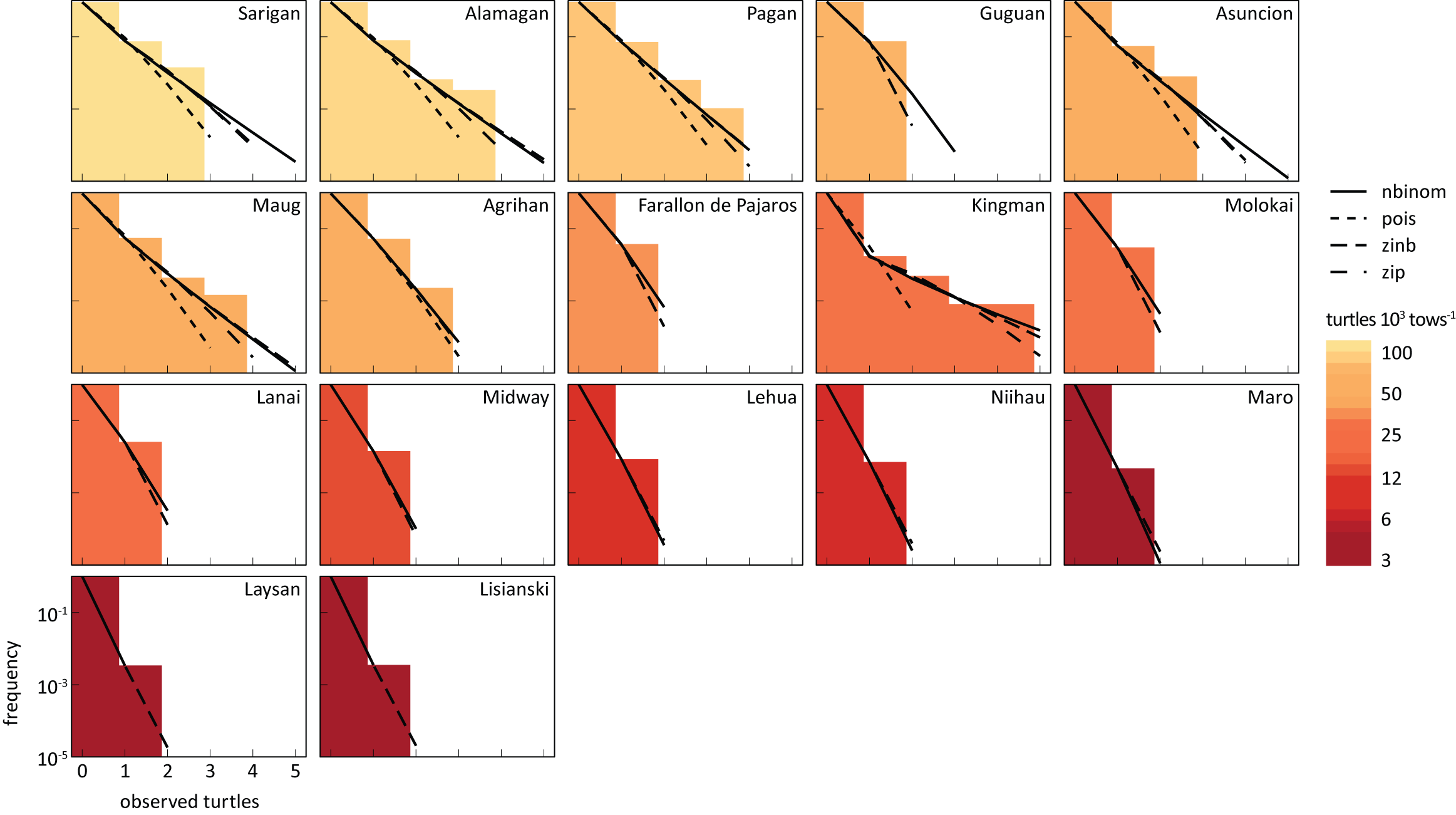
­

**Figure B.** **Count frequency histograms and model fits for all islands/atolls not included in the main manuscript.** The five sites with the greatest turtle densities for each of the four regions (Pacific Remote Island Areas, the Marianas, American Samoa, and Hawaii) were visualized within the main manuscript. The sites included here represent all other locations for which count models were produced. The four line types in each panel illustrate the comparative fits of negative binomial (nbinom), poisson (pois), zero-inflated negative binomial (zinb) and zero-inflated poisson (zip). Panels are ordered and histograms are shaded by predicted turtle density.


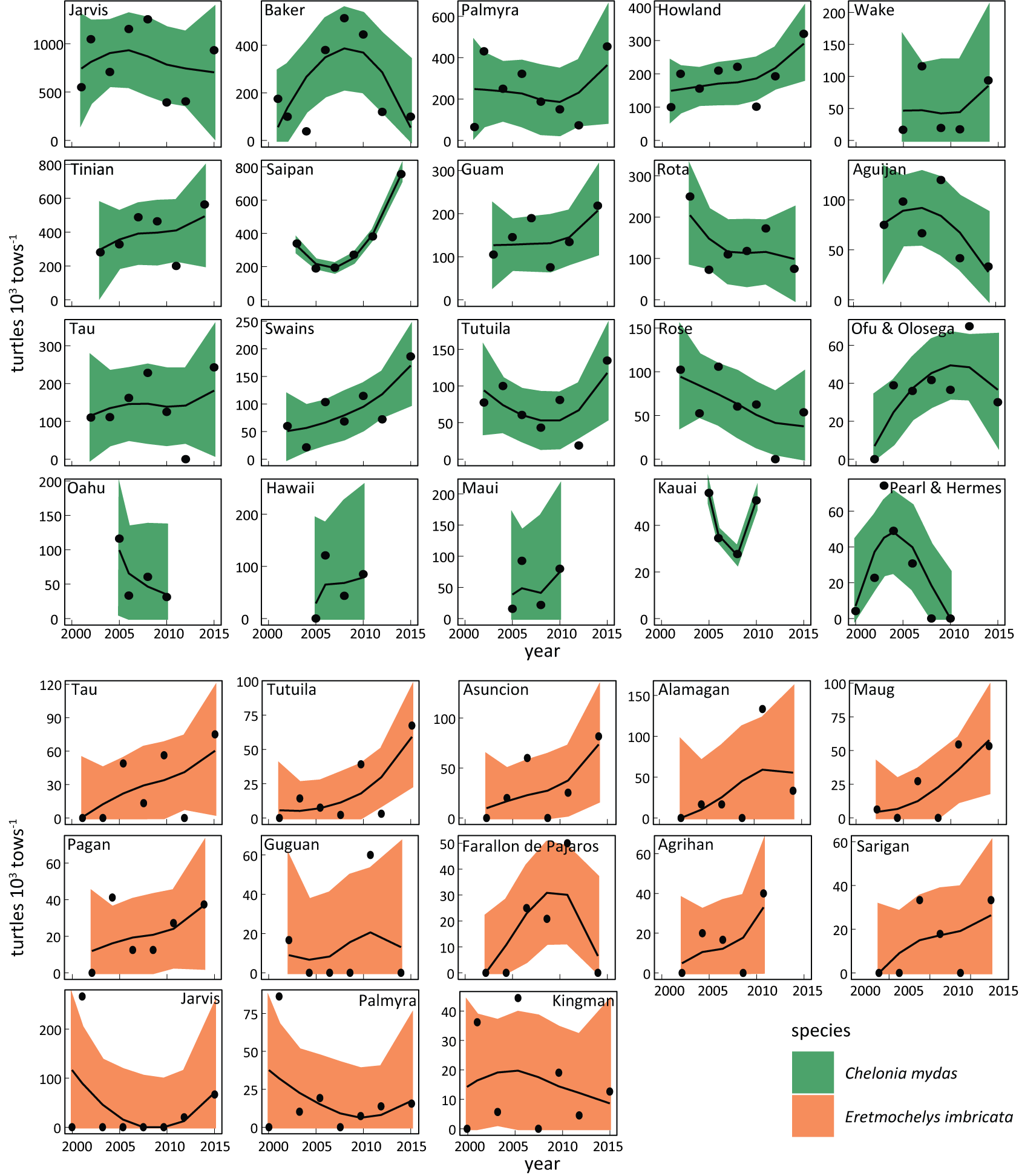


**Figure C.** **Island-scale yearly turtle densities show variable trends between survey locations and species.** Loess smoothed trends for green sea turtles (*Chelonia mydas*) appear either stable (albeit fluctuating year to year) or increasing. Saipan shows the most highly positive trend, with steady increases since 2005. Trends for hawksbills (*Eretmochelys imbricata*) are dominated by modest positive trends, but with much lower overall densities.

**
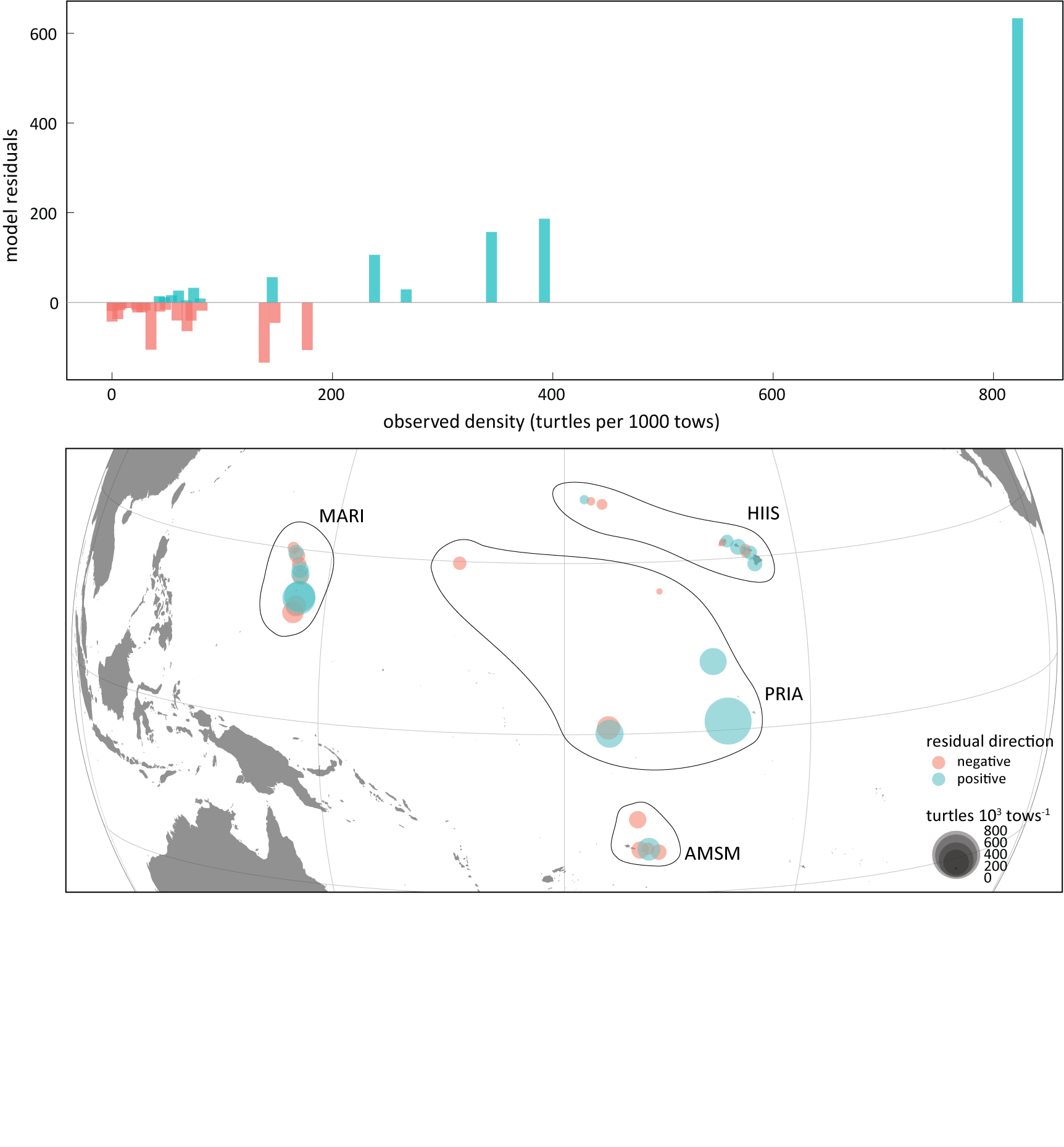
**

**Figure D.** **Random forest model contains a great deal of unexplained variation, particularly for islands with high turtle densities.** The upper panel shows random forest model residuals plotted against observed green turtle densities. The lower panel maps island locations, colored by positive versus negative residuals in accordance with the values shown on the upper panel, and with size determined by density. Sites are grouped by the four regions assessed: the Mariana Archipelago (MARI), the Hawaiian Archipelago (HIIS), the Pacific Remote Island Areas (PRIA) and American Samoa (AMSM). Jarvis Island, with an observed density of 822 green turtles per 1000 towed-diver transect segments, is the most prominent outlier.


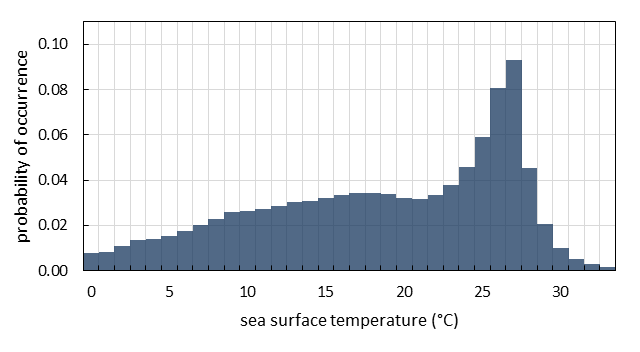


**Figure E**. **The climate envelope of 993 exploited marine fishes peaks at 27-28 °C, similar to our observation for sea turtle density (Fig 5A-B).** To understand this, we generated the parameters for the normal distribution of inferred temperature preference for 993 species of fishes exploited in global fisheries, given the 25^th^, 50^th^, and 75^th^ empirical quantiles provided in the supplemental material in Cheung, Watson, and Pauly (2013). Using these parameters, we summed the discrete preference probabilities in each 1 °C band, and plot the results from 0-35 °C. Blue columns represent the modeled probabilities in 1 °C bands. Cheung, Watson, and Pauly (2013) used each species thermal preference weighted by landings masses, to document the tropicalization of the global fisheries landings, likely reflecting a serial warming of ocean regions. Here, we simply use these data to corroborate our findings of a similar temperature preference, not for the geographic distribution of marine fishes, but for sea turtle densities.

**Table A.** **Density (turtles per 1000 tow segments and turtles per km) and predicted number of individuals per survey area.** Density is derived from negative binomial count models fit for 53 islands across the US Pacific surveyed via towed-diver surveys. Separate models and density estimates were fit for combined species (TOT), green turtles only (CM), and hawksbill turtles only (EI). Predicted individuals reflect density values multiplied by the number of transect tow segments completed for each island.

| **Survey location** | | **Turtles per 1000 tow segments** | | | **Turtles per km** | | | **Predicted individuals** | | |
| --- | --- | --- | --- | --- | --- | --- | --- | --- | --- | --- |
| **Region** | **Island/Atoll** | **TOT** | **CM** | **EI** | **TOT** | **CM** | **EI** | **TOT** | **CM** | **EI** |
| **Pacific Remote Island Areas** | Jarvis | 844 | 822 | 22 | 3.72 | 3.62 | 0.10 | 80 | 78 | 2 |
|  | Baker | 276 | 267 | 9 | 1.25 | 1.21 | 0.04 | 19 | 18 | 1 |
|  | Palmyra | 254 | 238 | 16 | 1.12 | 1.05 | 0.07 | 48 | 45 | 3 |
|  | Howland | 182 | 177 | 5 | 0.82 | 0.80 | 0.02 | 14 | 14 | 0 |
|  | Wake | 49 | 49 | 0 | 0.23 | 0.23 | 0.00 | 8 | 8 | 0 |
|  | Kingman | 30 | 14 | 16 | 0.13 | 0.06 | 0.07 | 5 | 2 | 3 |
|  | Johnston | 5 | 5 | 0 | 0.02 | 0.02 | 0.00 | 1 | 1 | 0 |
| **Marianas** | Tinian | 394 | 392 | 0 | 1.78 | 1.77 | 0.00 | 37 | 37 | 0 |
|  | Saipan | 344 | 344 | 0 | 1.60 | 1.60 | 0.00 | 50 | 50 | 0 |
|  | Guam | 149 | 148 | 0 | 0.65 | 0.65 | 0.00 | 34 | 33 | 0 |
|  | Rota | 138 | 138 | 0 | 0.64 | 0.64 | 0.00 | 14 | 14 | 0 |
|  | Sarigan | 112 | 98 | 14 | 0.55 | 0.48 | 0.07 | 5 | 5 | 1 |
|  | Alamagan | 103 | 80 | 23 | 0.49 | 0.38 | 0.11 | 5 | 4 | 1 |
|  | Pagan | 87 | 67 | 20 | 0.42 | 0.33 | 0.10 | 14 | 11 | 3 |
|  | Aguijan | 77 | 77 | 0 | 0.34 | 0.34 | 0.00 | 3 | 3 | 0 |
|  | Guguan | 75 | 59 | 16 | 0.38 | 0.30 | 0.08 | 3 | 2 | 1 |
|  | Asuncion | 71 | 45 | 26 | 0.34 | 0.22 | 0.12 | 4 | 3 | 2 |
|  | Maug | 68 | 47 | 20 | 0.33 | 0.23 | 0.10 | 8 | 5 | 2 |
|  | Agrihan | 57 | 43 | 14 | 0.28 | 0.21 | 0.07 | 5 | 4 | 1 |
|  | Farallon de Pajaros | 37 | 22 | 15 | 0.19 | 0.11 | 0.08 | 2 | 1 | 1 |
|  | Anatahan | 0 | 0 | 0 | 0.00 | 0.00 | 0.00 | 0 | 0 | 0 |
|  | Arakane | 0 | 0 | 0 | 0.00 | 0.00 | 0.00 | 0 | 0 | 0 |
|  | Pathfinder | 0 | 0 | 0 | 0.00 | 0.00 | 0.00 | 0 | 0 | 0 |
|  | Santa Rosa | 0 | 0 | 0 | 0.00 | 0.00 | 0.00 | 0 | 0 | 0 |
|  | Stingray | 0 | 0 | 0 | 0.00 | 0.00 | 0.00 | 0 | 0 | 0 |
|  | Supply | 0 | 0 | 0 | 0.00 | 0.00 | 0.00 | 0 | 0 | 0 |
|  | Tatsumi | 0 | 0 | 0 | 0.00 | 0.00 | 0.00 | 0 | 0 | 0 |
| **American Samoa** | Ta’u | 173 | 145 | 28 | 0.76 | 0.63 | 0.12 | 24 | 20 | 4 |
|  | Swains | 92 | 82 | 10 | 0.42 | 0.38 | 0.05 | 9 | 8 | 1 |
|  | Tutuila | 90 | 72 | 19 | 0.42 | 0.34 | 0.09 | 31 | 24 | 6 |
|  | Rose | 71 | 68 | 3 | 0.33 | 0.31 | 0.01 | 11 | 11 | 0 |
|  | Ofu & Olosega | 39 | 35 | 4 | 0.17 | 0.15 | 0.02 | 5 | 5 | 1 |
|  | South Bank | 0 | 0 | 0 | 0.00 | 0.00 | 0.00 | 0 | 0 | 0 |
| **Hawaii** | Oahu | 74 | 74 | 0 | 0.11 | 0.11 | 0.00 | 11 | 11 | 0 |
|  | Hawaii | 64 | 60 | 4 | 0.28 | 0.27 | 0.02 | 23 | 21 | 1 |
|  | Maui | 54 | 54 | 0 | 0.24 | 0.24 | 0.00 | 13 | 13 | 0 |
|  | Kauai | 43 | 43 | 0 | 0.18 | 0.18 | 0.00 | 8 | 8 | 0 |
|  | Molokai | 30 | 30 | 0 | 0.13 | 0.13 | 0.00 | 3 | 3 | 0 |
|  | Pearl & Hermes | 27 | 27 | 0 | 0.12 | 0.12 | 0.00 | 6 | 6 | 0 |
|  | Lanai | 26 | 23 | 0 | 0.11 | 0.10 | 0.00 | 3 | 2 | 0 |
|  | Kure | 18 | 18 | 0 | 0.08 | 0.08 | 0.00 | 2 | 2 | 0 |
|  | Midway | 14 | 14 | 0 | 0.06 | 0.06 | 0.00 | 2 | 2 | 0 |
|  | Lehua | 8 | 8 | 0 | 0.04 | 0.04 | 0.00 | 0 | 0 | 0 |
|  | Niihau | 7 | 7 | 0 | 0.03 | 0.03 | 0.00 | 1 | 1 | 0 |
|  | Maro | 4 | 4 | 0 | 0.02 | 0.02 | 0.00 | 0 | 0 | 0 |
|  | French Frigate | 3 | 3 | 0 | 0.01 | 0.01 | 0.00 | 0 | 0 | 0 |
|  | Laysan | 3 | 3 | 0 | 0.01 | 0.01 | 0.00 | 0 | 0 | 0 |
|  | Lisianski | 3 | 3 | 0 | 0.01 | 0.01 | 0.00 | 0 | 0 | 0 |
|  | Gardner | 0 | 0 | 0 | 0.00 | 0.00 | 0.00 | 0 | 0 | 0 |
|  | Kaula | 0 | 0 | 0 | 0.00 | 0.00 | 0.00 | 0 | 0 | 0 |
|  | Necker | 0 | 0 | 0 | 0.00 | 0.00 | 0.00 | 0 | 0 | 0 |
|  | Nihoa | 0 | 0 | 0 | 0.00 | 0.00 | 0.00 | 0 | 0 | 0 |
|  | Raita | 0 | 0 | 0 | 0.00 | 0.00 | 0.00 | 0 | 0 | 0 |

**Table B.** **∆AIC values for towed-diver survey observations for each study location fit with four candidate models: negative binomial (nbinom), zero-inflated negative binomial (ZINB), poisson (pois), and zero-inflated poisson (ZIP).** Several islands were excluded as the number of total observations were too low to successfully fit one or more of these models. Lowest AIC values indicate the best fit model. ∆AIC must be > 3 to determine superior fit; ∆AIC ≤3 renders the models indistinguishable. Nbinom most often showed the lowest AIC, and ∆AIC was always < 2.4, and therefore is the most appropriate of these four models for this dataset.

| **Survey location** | | **Model ∆AIC** | | | |
| --- | --- | --- | --- | --- | --- |
| **Region** | **Island/Atoll** | **NegBin** | **ZINB** | **Poisson** | **ZIP** |
| **American Samoa** | Ofu & Olosega | 0.0 | 1.9 | 14.7 | 0.0 |
|  | Rose | 0.0 | 1.4 | 29.6 | 8.4 |
|  | Swains | 0.0 | 1.8 | 107.8 | 14.0 |
|  | Tau | 0.0 | 2.0 | 517.5 | 64.5 |
|  | Tutuila | 1.1 | 0.0 | 225.0 | 90.4 |
| **Marianas** | Agrihan | 1.9 | 3.9 | 0.0 | 1.9 |
|  | Aguijan | 0.0 | 1.9 | 29.0 | 5.7 |
|  | Alamagan | 0.0 | 1.9 | 1.8 | 0.5 |
|  | Asuncion | 0.3 | 2.0 | 1.3 | 0.0 |
|  | Farallon de Pajaros | 2.0 | 3.7 | 0.0 | 2.0 |
|  | Guam | 0.0 | 0.8 | 170.7 | 34.6 |
|  | Guguan | 2.0 | 2.7 | 0.0 | 2.0 |
|  | Maug | 0.0 | 1.9 | 4.0 | 0.5 |
|  | Pagan | 0.0 | 2.0 | 3.2 | 0.3 |
|  | Rota | 0.0 | 1.9 | 59.7 | 7.5 |
|  | Saipan | 0.0 | 1.8 | 219.5 | 34.4 |
|  | Sarigan | 0.5 | 2.1 | 1.7 | 0.0 |
|  | Tinian | 0.0 | 0.4 | 184.3 | 41.9 |
| **Hawaii** | Hawaii | 0.0 | 1.9 | 66.7 | 4.5 |
|  | Kauai | 2.1 | 2.1 | 41.9 | 0.0 |
|  | Lanai | 2.0 | 3.8 | 0.0 | 2.0 |
|  | Lehua | 2.0 | 4.0 | 0.0 | 2.0 |
|  | Maui | 0.0 | 2.0 | 42.6 | 1.4 |
|  | Molokai | 2.0 | 3.8 | 0.0 | 2.0 |
|  | Niihau | 2.0 | 4.0 | 0.0 | 2.0 |
|  | Oahu | 0.0 | 2.0 | 6.9 | 0.2 |
|  | Laysan | 2.0 | 4.0 | 0.0 | 2.0 |
|  | Lisianski | 2.0 | 4.0 | 0.0 | 2.0 |
|  | Maro | 2.0 | 4.0 | 0.0 | 2.0 |
|  | Midway | 2.0 | 3.9 | 0.0 | 2.0 |
|  | Pearl & Hermes | 0.0 | 2.0 | 41.8 | 4.8 |
| **Pacific Remote Island Areas** | Baker | 0.0 | 1.8 | 156.9 | 31.2 |
|  | Howland | 0.0 | 1.3 | 15.8 | 3.9 |
|  | Jarvis | 2.4 | 0.0 | 637.2 | 217.6 |
|  | Kingman | 0.0 | 1.8 | 48.1 | 0.3 |
|  | Palmyra | 0.1 | 0.0 | 518.7 | 129.6 |
|  | Wake | 0.0 | 3.8 | 14.5 | 2.1 |

| **Survey location** | | | **Survey effort** | | | | |
| --- | --- | --- | --- | --- | --- | --- | --- |
| **Region** | **Site** | **Site type** | **Dates** | **Years** | **Tows** | **Segments** | **Turtles** |
| **American Samoa** | Tutuila |  | 47 | 7 | 249 | 2399 | 217 |
|  | Tau |  | 18 | 7 | 97 | 959 | 167 |
|  | Rose |  | 23 | 7 | 117 | 1145 | 81 |
|  | Swains |  | 18 | 7 | 73 | 710 | 65 |
|  | Ofu & Olosega |  | 19 | 7 | 98 | 964 | 38 |
|  | South Bank |  | 1 | 1 | 6 | 36 | 0 |
| **Marianas** | Saipan |  | 18 | 6 | 90 | 880 | 303 |
|  | Tinian |  | 12 | 6 | 58 | 566 | 223 |
|  | Guam |  | 27 | 6 | 139 | 1366 | 204 |
|  | Rota |  | 12 | 6 | 63 | 630 | 87 |
|  | Pagan |  | 18 | 6 | 96 | 954 | 83 |
|  | Maug |  | 16 | 6 | 70 | 695 | 47 |
|  | Sarigan |  | 6 | 6 | 30 | 295 | 33 |
|  | Alamagan |  | 6 | 6 | 30 | 300 | 31 |
|  | Asuncion |  | 8 | 6 | 38 | 378 | 27 |
|  | Agrihan |  | 8 | 5 | 44 | 438 | 25 |
|  | Aguijan |  | 6 | 6 | 26 | 258 | 20 |
|  | Guguan |  | 6 | 6 | 26 | 254 | 19 |
|  | Anatahan |  | 2 | 1 | 12 | 120 | 13 |
|  | Farallon de Pajaros |  | 7 | 6 | 27 | 268 | 10 |
|  | Arakane |  | 2 | 2 | 9 | 87 | 1 |
|  | Pathfinder |  | 2 | 2 | 7 | 69 | 0 |
|  | Santa Rosa |  | 2 | 2 | 6 | 58 | 0 |
|  | Stingray |  | 1 | 1 | 4 | 30 | 0 |
|  | Supply |  | 1 | 1 | 1 | 6 | 0 |
|  | Tatsumi |  | 1 | 1 | 2 | 19 | 0 |
| **Hawaii** | Hawaii |  | 28 | 4 | 147 | 1425 | 91 |
|  | Maui |  | 20 | 4 | 99 | 965 | 52 |
|  | Oahu |  | 14 | 4 | 61 | 580 | 43 |
|  | Kauai |  | 14 | 4 | 78 | 746 | 32 |
|  | Lanai |  | 8 | 4 | 43 | 430 | 11 |
|  | Molokai |  | 8 | 4 | 37 | 367 | 11 |
|  | Niihau |  | 11 | 4 | 45 | 424 | 3 |
|  | Kaula |  | 1 | 1 | 3 | 27 | 1 |
|  | Lehua |  | 5 | 4 | 12 | 118 | 1 |
|  | Pearl & Hermes |  | 35 | 7 | 179 | 1764 | 48 |
|  | Kure |  | 21 | 7 | 95 | 947 | 17 |
|  | Midway |  | 17 | 5 | 78 | 774 | 11 |
|  | French Frigate |  | 32 | 8 | 140 | 1358 | 4 |
|  | Maro |  | 22 | 7 | 91 | 901 | 4 |
|  | Lisianski |  | 21 | 7 | 89 | 876 | 3 |
|  | Laysan |  | 6 | 6 | 31 | 296 | 1 |
|  | Gardner |  | 3 | 3 | 5 | 50 | 0 |
|  | Necker |  | 3 | 3 | 12 | 104 | 0 |
|  | Nihoa |  | 1 | 1 | 3 | 25 | 0 |
|  | Raita |  | 3 | 1 | 5 | 41 | 0 |
| **Pacific Remote Island Areas** | Jarvis |  | 17 | 8 | 79 | 764 | 645 |
|  | Palmyra |  | 34 | 8 | 157 | 1520 | 386 |
|  | Baker |  | 15 | 8 | 58 | 562 | 155 |
|  | Howland |  | 16 | 8 | 65 | 615 | 112 |
|  | Kingman |  | 28 | 8 | 143 | 1410 | 42 |
|  | Wake |  | 20 | 5 | 88 | 843 | 41 |
|  | Johnston |  | 26 | 6 | 121 | 1143 | 6 |

**Table C. Survey effort by location.** Summary values for number of days, years, tows, tow-segments, and turtles counted across all surveys for each study site.
